# Mobile Health App as an Auxiliary Tool in Management of Atopic Dermatitis in Children: Randomized Controlled Trial

**DOI:** 10.2196/60479

**Published:** 2025-01-22

**Authors:** Alex Zvulunov, Stepan Lenevich, Natalia Migacheva

**Affiliations:** 1 Sheba Medical Center and the Recanati School of Medicine, Reichman University, Tel Hashomer, Ramat-Gan, Israel, 972 584440189; 2 AvantaTrading Ltd, Moscow, Russian Federation; 3 Department of Pediatrics, Samara State Medical University, Samara, Russian Federation

**Keywords:** atopic dermatitis, skin, disease management, children, pediatric, feasibility, mHealth, mobile health, app, eczema, Atopic App, dermatology

## Abstract

**Background:**

Mobile health apps can boost treatment adherence and support disease management at home. The Atopic App and web-based Atopic School patient education program offer a chance to enhance adherence to atopic dermatitis (AD) management.

**Objective:**

We aim to evaluate the feasibility, acceptability, and preliminary efficacy of the Atopic App mobile health intervention in the managing of AD in children.

**Methods:**

A randomized controlled study in children with AD divided participants into 3 groups: a control group (no app), an observational group with the app, and an interventional group with investigator supervision. Patients were examined at screening and follow-up visits 1 and 2 at 3-month intervals. Outcome measures included SCORAD (Scoring Atopic Dermatitis) for objective severity and Patient-Oriented Eczema Measure (POEM) for subjective effectiveness. Statistical analysis used paired *t* tests (2-tailed), the Mann-Whitney U test, and multiple regression.

**Results:**

Fifty-eight participants entered this study (38 boys and 20 girls): group 1 (control) comprised 17 patients, while experimental groups 2 and 3 consisted of 20 and 21 patients, respectively. The rates of missed appointments were similar and statistically insignificant across the groups. All groups showed a significant decrease in SCORAD and POEM scores (*P*<.05). Usage of the app for ≥8 days showed a more significant decrease in severity scores compared to those who used it for ≤7 days, or did not use it at all. Participants who used the app for ≥8 days had a median SCORAD of 6.25 (95% CI 4.6‐14.1; IQR 4-16.3) at visit 1, significantly lower than nonusers (17.9, 95% CI 13.9‐24.0; IQR 13.9-24; *P*=.03) and those using it ≤7 days (13, 95% CI 9.35‐27; IQR 7.2-27; *P*=.04). Their median POEM of 2 (95% CI 1.0‐4.5; IQR 1-5.3) was also significantly lower than those using the app ≤7 days (9, 95% CI 2‐12; IQR 2-12; *P*=.04) and lower, though not significantly, than nonusers (7, 95% CI 1‐9; IQR 1-9; *P*=.14). Additionally, using the Atopic App for ≥8 days after the screening visit strongly predicted a decrease in both SCORAD and POEM scores (*P*=.01 and *P*=.04, respectively). The time since the screening visit significantly predicted increased outcome scores, while prescriptions of topical calcineurin inhibitors, oral antihistamines, and oral antibiotics were weak and insignificant predictors of score changes.

**Conclusions:**

Our findings indicate that the Atopic App is helpful tool in managing AD in children, and they underscore the potential of mobile health interventions in the disease management.

## Introduction

Poor medication adherence is a major barrier to treatment success in atopic dermatitis (AD), due to various underlying causes, including forgetfulness, medication side effects, complex dosing regimens, cost barriers, etc [1]. Mobile health apps may improve treatment adherence [2]. There is a growing list of AD-related mobile apps. A recent systematic review and meta-analysis of mobile health applications for AD reported a significant improvement in patients’ quality of life (assessed by Dermatology Life Quality Index) and self-management (assessed by Patient-Oriented Eczema Measure [POEM]) but no significant impact on AD severity (assessed by SCORAD [Scoring Atopic Dermatitis]) [3]. POEM is a subjective measure completed by patients, capturing their experience of AD severity (scores range from 0‐28, higher scores indicate worse symptoms). SCORAD is a clinician-administered tool that combines objective assessment of disease signs with patient-reported symptoms (scores range from 0‐103, higher scores indicate worse severity). The most important feature required for development of mobile apps for caregivers of children with AD is an educational functionality including knowledge of the disease, management of symptoms, medication usage, and triggers [4]. Most available apps for AD primarily assess disease severity, lacking educational functionality or bidirectional communication. Few have been scientifically studied, mainly demonstrating feasibility [3-5].

The Atopic App is a free to download app on the App Store (Apple Inc) and Google Play (Google LLC) that address these shortcomings by offering the following features: (1) a chatbot-directed instruction on app use and targeted education to enhance user understanding and adherence to treatment plans, (2) online patient-education program beyond basic disease information, (3) automatic artificial intelligence (AI)-powered severity assessment for efficient self-monitoring by patients and caregivers, (4) integration of personalized action plans prescribed by health care providers, and (5) a tool for identification of personal trigger factors to reduce flare-ups. The engagement process with the app includes completion of the POEM questionnaire, acquisition of clinical photographs and numerical rating scale for severity of itch, transcription of action plans prescribed by a treating physician, documentation of suspected triggers of exacerbations, and the patient education Atopic School program, while an integrated AI tool automatically calculates severity scores using the Eczema Area and Severity Index method based on photographs taken by users [6]. Recently, we reported on feasibility and impact of the Atopic App that provided a real-world data on severity dynamics, treatment patterns and exacerbation-trigger correlations, indicating the tool’s potential impact on health care engagement of AD in children [6].

The purpose of this randomized controlled study is to evaluate the impact of the Atopic App mobile health app as an auxiliary management tool for children with AD.

## Methods

### Study Design

Study participants were children with AD aged 4 months till 16 years and their parents, consecutively recruited from our dermatology clinic regardless of gender, or disease severity. Informed consent was obtained from all participating parents.

This study used a parallel, 3-arm randomized controlled trial design with a 1:1:1 allocation ratio. Participants were randomized to 1 of 3 groups: a control group that did not use the Atopic App (group 1), an experimental observational group provided with the mobile app without supervision by the investigators (group 2), and an experimental interventional group provided with the mobile app with potential supervision by the investigators (group 3). To ensure allocation concealment and minimize selection bias, a sequential allocation to a study group was used. Upon study enrollment, participants received recommended treatment plans and instructions for contacting the doctor via messenger for any questions during treatment. Furthermore, participants in groups 2 and 3 were instructed to download the mobile app within 1 day post screening, while those in group 3 were also informed about the doctors’ virtual oversight, including registration status and regularity of use of the app. Patients aged older than 14 years were permitted to use the app. Beyond this, no specific instructions or recommendations were given regarding usage of the Atopic App, allowing participants to access its features and functionalities as needed to manage their children’s AD. Push notifications served as reminders to submit a POEM form, initially after 7 days and then daily until completion of participation in this study.

The investigators did not initiate communication or reminders regarding usage of the app, but could refer to usage data reports during patients’ visits or queries. The WhatsApp (Meta Platforms) application was used for patient-doctor communication. Reasons for communications and for interim visits were registered by the following categories: exacerbation, lack of improvement, or clarification questions. Whenever adjustments to therapy were deemed necessary due to ineffectiveness or exacerbations, patients were invited for an in-person interim visit.

Patient-parent pairs were excluded if they had previous experience with the Atopic App or participation in affiliated online Atopic School program or presence of concomitant skin disease or pathological conditions that may affect the assessment of effectiveness (severe somatic diseases, mental disorders, oncologic or acute infectious diseases, etc) or, regarding participants in groups 2 or 3, avoidance from registration during consecutively 5 days following the screening visit. In addition, participants were excluded from the final analysis, if the time gap between their visits deviated by more than 30 days from the scheduled dates, either by occurring more than 30 days before or exceeding 30 days after the planned follow-up visit. This exclusion criterion resulted in variations in the number of patients across different stages of this study within the groups.

The intended duration of this study was 6 months with 3 months intervals between visits.

The outcome end points included objective severity assessment using the SCORAD scale, and subjective assessment of effectiveness using the POEM scale.

To evaluate the significance of pairwise differences among the groups under consideration, the Mann-Whitney U test was used. Additionally, multiple regression analysis was used to explore relationships between POEM and SCORAD score changes and independent variables such as the prescription of different types of medications at the previous visit, the period of time since the screening visit, and whether the patient engaged with the Atopic App for 8 and more days following the screening visit.

To evaluate the impact of usage of the Atopic App on AD severity dynamics, participants were stratified into 3 engagement groups. Group A included control group participants who did not install the app, group B included participants from both experimental groups who used the app 7 days or less between screening and visit 1 or 2, and group C included participants from both experimental groups who used the app 8 or more days between screening and visits 1 or 2.

### Ethical Considerations

This study was approved by the Samara State Medical University Ethics Committee (review 242). Written informed consent was obtained, detailing data usage, potential risks, and the right to withdraw at any time. Comprehensive safety and security procedures were implemented to protect participant privacy and reduce harm. Data transmission was encrypted using HTTPS, and participant data were anonymized with unique codes. Staff were trained on data security protocols and privacy regulations. No compensation was provided to participants. Due to the pilot nature of this study and its limited sample size, registration in a World Health Organization–accredited registry was not conducted before this study. However, it was registered retrospectively (ClinicalTrials.gov NCT06412094).

## Results

**Figure 1.**
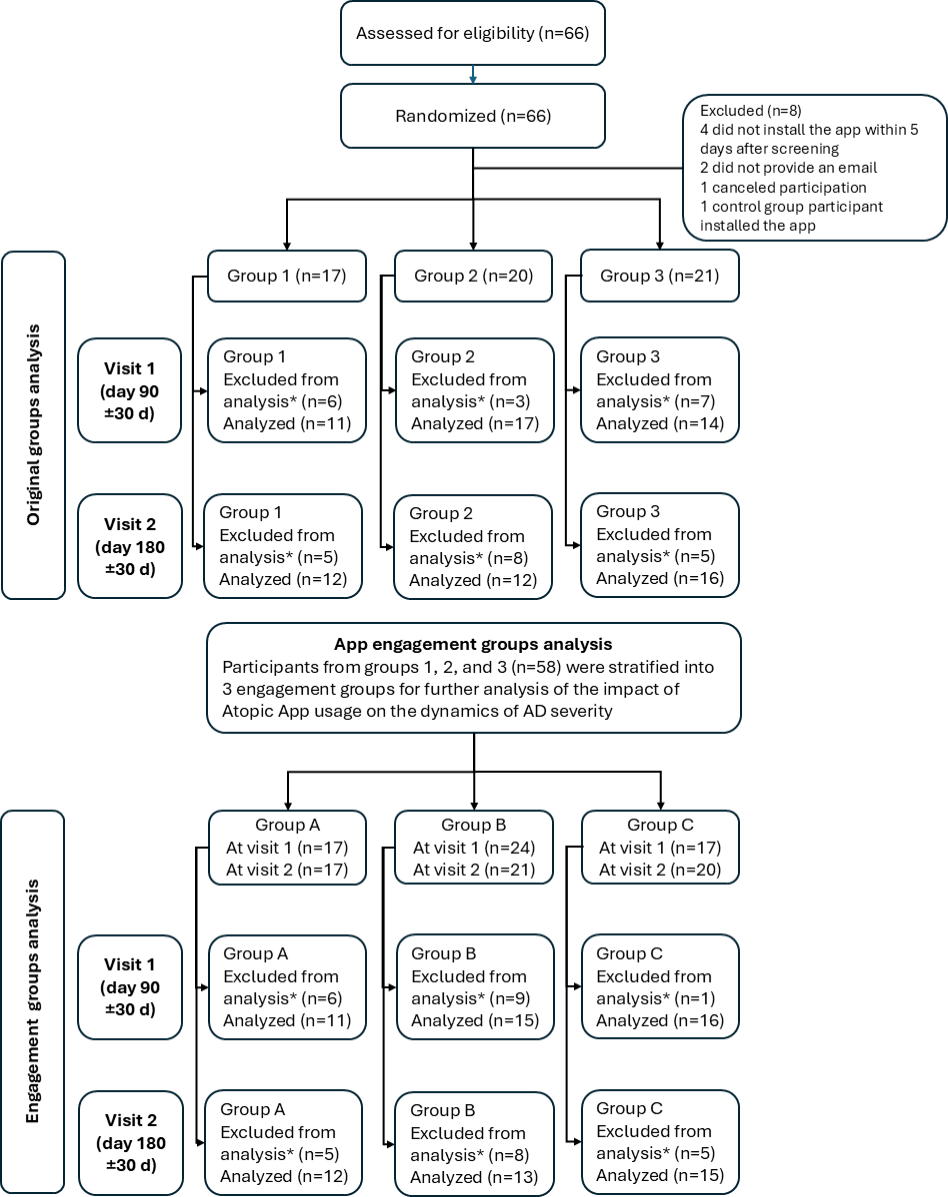
CONSORT flow diagram. *Patients who missed follow-up appointments beyond the ±30 day window were excluded from the analysis. AD: atopic dermatitis; CONSORT: Consolidated Standards of Reporting Trials.

During the period from March 2022 till June 2022, a total of 66 children with AD and their parents were recruited for this study. Seven patients from experimental groups 2 and 3 were excluded after the screening: those who did not install the app on time, those who did not provide their email address, or those who cancelled their participation after the screening. Moreover, 1 participant from the control group 1 was excluded due to installation of the app during this study. So, 58 participants entered this study (38 boys and 20 girls): group 1 (control) comprised 17 patients, while experimental groups 2 and 3 consisted of 20 and 21 patients, respectively. Flow of participants through each stage of the study (enrollment, intervention allocation, follow-up, and analysis) is depicted in the Figure 1. Baseline demographic data and clinical features in each group are presented in the Table 1.

**Table 1.** Baseline demographic data and clinical features in each study group.

		Group 1 (n=17)	Group 2 (n=20)	Group 3 (n=21)
**Age (years)**				
	Median (IQR)	4.4 (1.1‐6.2)	2.1 (0.6‐8)	7.2 (1.5‐11.3)
	Range	0.5‐15	0.3‐16.3	0.5‐14.1
Male, n		13	13	12
Female, n		4	7	9
**SCORAD^a^**				
	Median (IQR)	31.8 (24.5‐38.35)	31.4 (24.5‐38.2)	34 (22.7‐43.6)
	Range	8‐61	12‐50.9	9.4‐49
**POEM^b^**				
	Median (IQR)	14 (8.5‐18.5)	12.5 (8.25‐16)	13 (9-16)
	Range	2‐23	5‐26	3‐20
Topical corticosteroids, n (%)		11 (65)	14 (70)	10 (48)
Topical calcineurin inhibitor, n (%)		10 (59)	16 (80)	9 (43)
Oral antihistamines, n (%)		11 (65)	16 (80)	11 (52)
Oral antibiotics, n (%)		1 (6)	2 (10)	0 (0)

a SCORAD: Scoring Atopic Dermatitis.

b POEM: Patient-Oriented Eczema Measure.

The vast majority of this study’s cohort (48/58, 83%) had a documented history of allergic diseases: 66% (38/58) exhibited concurrent food allergies, bronchial asthma, and allergic rhinitis or a combination of these. Overall, 29% (17/58) exhibited mild AD, while 66% (38/58) presented with moderate to severe AD, and the remaining 5% (3/58) had severe AD. No statistically significant differences were found between patients of different groups by gender, age, and severity of the disease at the time of inclusion in this study.

At the screening visit, group 3 had higher median AD severity scores (POEM and SCORAD) compared to groups 1 and 2. The difference in SCORAD and POEM between the groups at screening was not statistically significant (Table 1).

Patients who missed follow-up appointments outside the ±30 day window were excluded from the analysis, resulting in variations in the number of participants between visits. The rates of missed appointments were similar and statistically insignificant across the groups.

**Figure 2.**
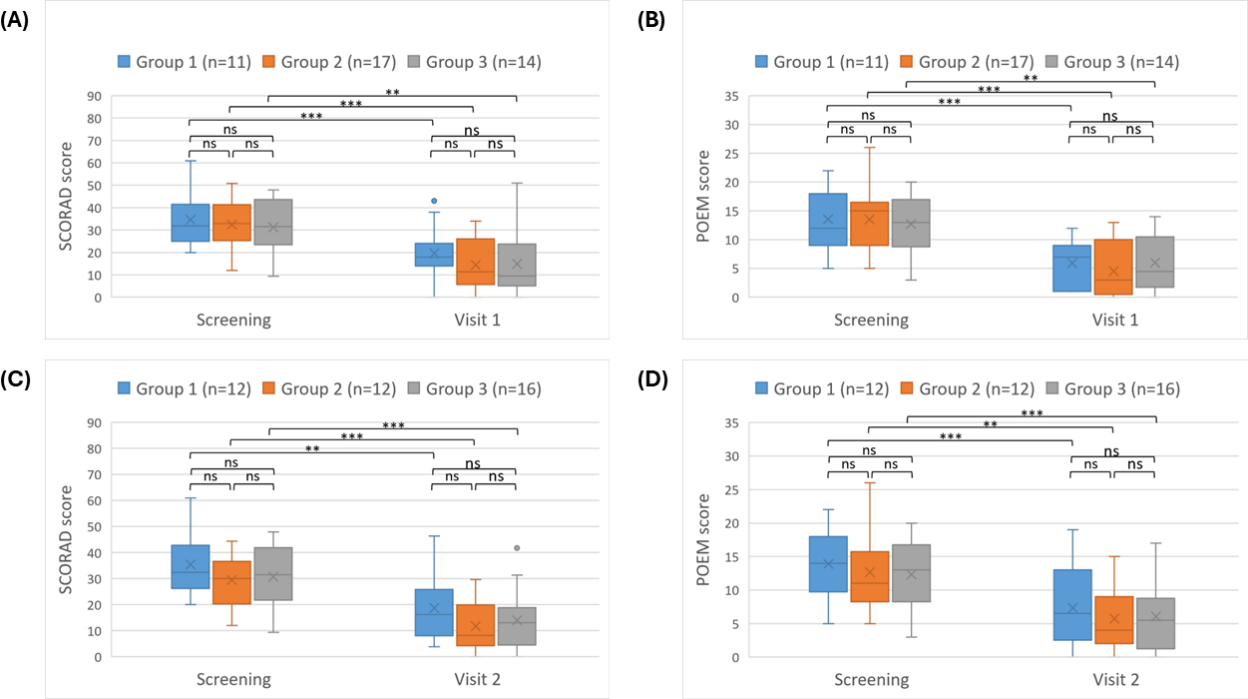
Distribution of AD severity scores across groups 1, 2, and 3. The error bars represent the minimum and maximum values excluding outliers. Above the bars, the intergroup statistical analysis is reported as follows: * *P*<.05, ** *P*<.01, *** *P*<.001. (A) SCORAD at screening and visit 1 (day 90±30 d). (B) POEM at screening and visit 1 (day 90±30 d). (C) SCORAD at screening and visit 2 (day 180±30 d). (D) POEM at screening and visit 2 (day 180±30 d). AD: atopic dermatitis; ns: not significant; POEM: Patient-Oriented Eczema Measure; SCORAD: Scoring Atopic Dermatitis.

Throughout the observation period, all groups demonstrated a significant decrease in the values of SCORAD and POEM scores at visit 1 and visit 2 (Figure 2) compared to corresponding scores at the screening visit (*P*<.05)

At visit 1, the median SCORAD scores for groups 2 and 3 were lower than group 1, at 11.3 (95% CI 6‐25) and 9.5 (95% CI 5.5‐20), respectively, compared to 17.9 (95% CI 13.9‐24) for group 1. At visit 2, this pattern continued, with medians of 8.20 (95% CI 4.85‐19.15) and 13 (95% CI 4.5‐18) for groups 2 and 3, respectively, versus 16.3 (95% CI 10.2‐24.9) for group 1. POEM scores followed a similar pattern. At visit 1, the median POEM scores for groups 2 and 3 were 3 (95% CI 1‐8) and 4.5 (95% CI 2‐9.5), respectively, compared to 7 (95% CI 1‐9) for group 1. At visit 2, the medians were 4 (95% CI 2‐9) for group 2 and 5.5 (95% CI 2‐8) for group 3, versus 6.5 (95% CI 3‐12) for group 1.

**Figure 3.**
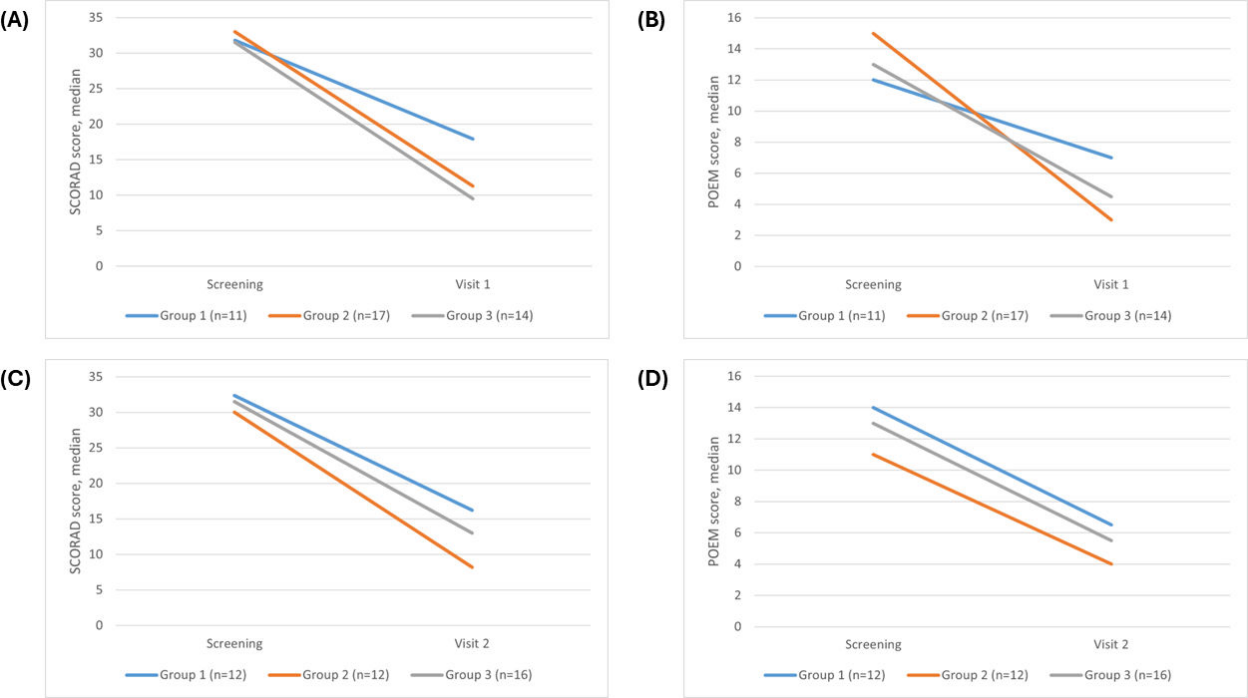
Trends in median AD severity scores across groups 1, 2, and 3. (A) Median SCORAD scores from screening to visit 1 (day 90±30 d). (B) Median POEM scores from screening to visit 1 (day 90±30 d). (C) Median SCORAD scores from screening to visit 2 (day 180±30 d). (D) Median POEM scores from screening to visit 2 (day 180±30 d). AD: atopic dermatitis; POEM: Patient-Oriented Eczema Measure; SCORAD: Scoring Atopic Dermatitis.

The reduction of SCORAD and POEM scores was more prominent and consistent for patients in groups 2 and 3 than in group 1, although the differences between the groups were not statistically significant (Table 2 and Figure 3).

**Table 2.** Median values of SCORAD^a^ and POEM^b^ scores across groups 1, 2, and 3 and visits.

		SCORAD, median (IQR)		POEM, median (IQR)	
		Screening	Visit	Screening	Visit
**Visit 1: day 90 (±30 d)**					
	Group 1^c^	31.8 (25‐41.5)	17.9 (13.9‐24)	12 (9-18)	7 (1-9)
	Group 2^d^	33 (25.3‐41.3)	11.3 (5.75‐26)	15 (9‐16.5)	3 (0.5‐10)
	Group 3^e^	31.5 (23.4‐43.6)	9.5 (5.05‐23.8)	13 (8.75‐17)	4.5 (1.75‐10.5)
**Visit 2: day 180 (±30 d)**					
	Group 1	32.4 (26.2‐42.8)	16.3 (8.1‐25.9)	14 (9.8‐18)	6.5 (2.5‐13)
	Group 2	30 (20.3‐36.6)	8.2 (4.3‐19.9)	11 (8.3‐15.8)	4 (2-9)
	Group 3	31.5 (21.8‐41.9)	13 (4.5‐18.9)	13 (8.3‐16.8)	5.5 (1.3‐8.8)

a SCORAD: Scoring Atopic Dermatitis.

b POEM: Patient-Oriented Eczema Measure.

c Group 1: control group that did not use the Atopic App.

d Group 2: experimental observational group provided with the mobile app without supervision by the investigators.

e Group 3: experimental interventional group provided with the mobile app with potential supervision by the investigators.

To further evaluate the effectiveness of using the Atopic App mobile health app to monitor the course of AD, an analysis was carried out considering the influence of engagement with the mobile application on treatment outcomes.

**Figure 4.**
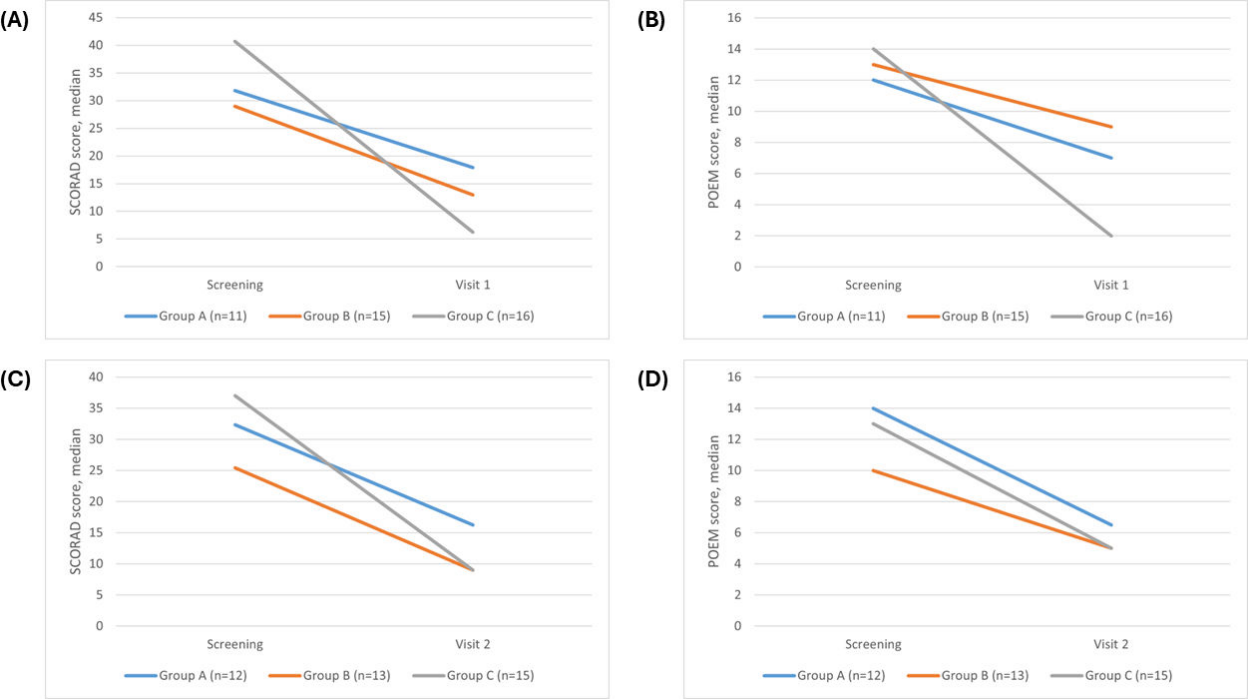
Trends in median AD severity scores across groups A, B, and C. (A) Median SCORAD scores from screening to visit 1 (day 90±30 d). (B) Median POEM scores from screening to visit 1 (day 90±30 d). (C) Median SCORAD scores from screening to visit 2 (180±30 d). (D). Median POEM scores from screening to visit 2 (day 180±30 d). AD: atopic dermatitis; POEM: Patient-Oriented Eczema Measure; SCORAD: Scoring Atopic Dermatitis.

At visit 1, the median SCORAD score of group C (6.25, 95% CI 4.6‐14.1) was significantly lower than that of both group A (17.9, 95% CI 13.9‐24; *P*=.03, *r*=0.418) and group B (13, 95% CI 9.35‐27; *P*=.04, *r*=0.369). The median POEM score of group C (2, 95% CI 1‐4.5) was significantly lower than that of group B (9, 95% CI 2‐12; *P*=.04, *r*=0.369) and lower than that of group A (7, 95% CI 1‐9), although this difference was not statistically significant (*P*=.14, *r*=0.285). At visit 2, despite a more pronounced reduction trend in group C (Figure 4; SCORAD 9, 95% CI 6‐18; POEM 5, 95% CI 2‐9), there were no statistically significant differences compared to group A (SCORAD 16.25, 95% CI 10.5‐24.9; POEM 6.5, 95% CI 3‐12) or group B (SCORAD 9, 95% CI 3.5‐17.5; POEM 5, 95% CI 1‐8).

The decrease in SCORAD and POEM scores was more significant in patients who used the app for 8 or more days as compared to those who used it for 7 days or less or did not use the app at all (Figures4  5; Table 3).

**Figure 5.**
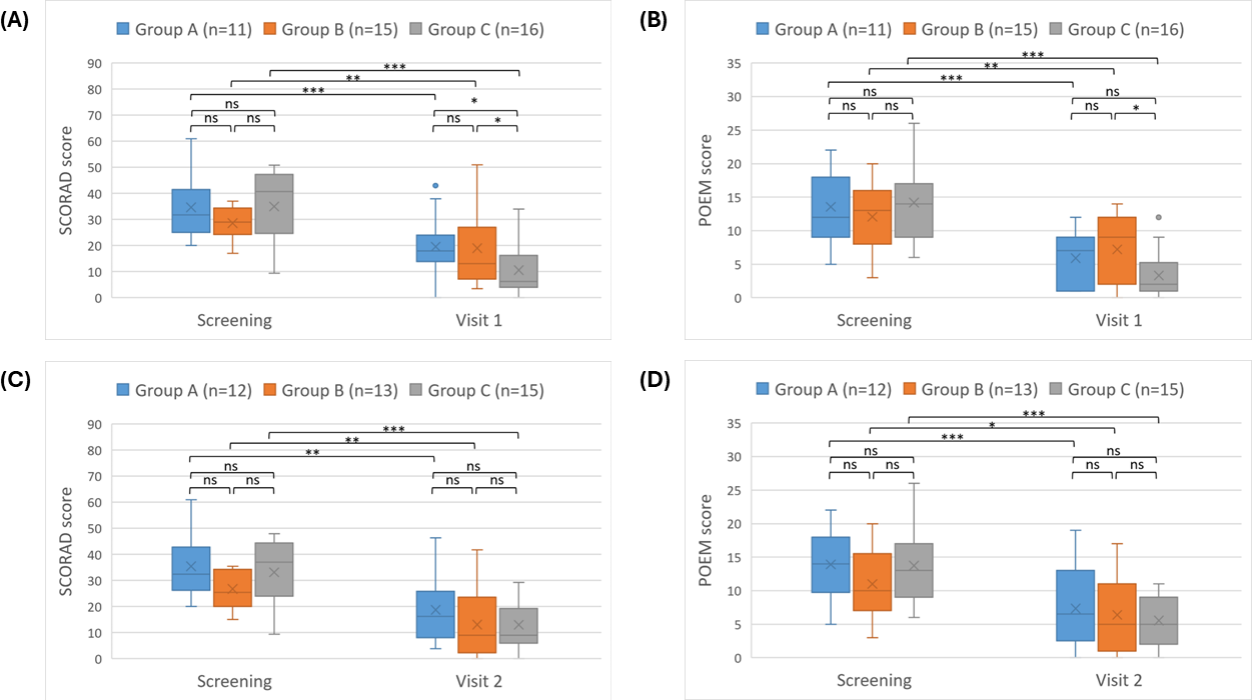
Distribution of AD severity scores across groups A, B, and C. The error bars represent the minimum and maximum values excluding outliers. Above the bars, the intergroup statistical analysis is reported as follows: * *P*<.05, ** *P*<.01, *** *P*<.001. (A) SCORAD at screening and visit 1 (day 90±30 d). (B) POEM at screening and visit 1 (day 90±30 d). (C) SCORAD at screening and visit 2 (day 180±30 d). (D) POEM at screening and visit 2 (day 180±30 d). AD: atopic dermatitis; POEM: Patient-Oriented Eczema Measure; SCORAD: Scoring Atopic Dermatitis.

**Table 3.** Median values of SCORAD^a^ and POEM^b^ scores.

		SCORAD, median (IQR)		POEM, median (IQR)	
		Screening	Visit	Screening	Visit
**Visit 1: day 90 (±30 d)**					
	Group A^c^	31.8 (25‐41.5)	17.9 (13.9‐24)	12 (9-18)	7 (1-9)
	Group B^d^	29 (24.2‐34.4)	13 (7.2‐27)	13 (8-16)	9 (2-12)
	Group C^e^	40.7 (24.6‐47.3)	6.25 (4‐16.3)	14 (9-17)	2 (1‐5.3)
**Visit 2: day 180 (±30 d)**					
	Group A	32.4 (26.3‐42.8)	16.3 (8.1‐25.9)	14 (9.8‐18)	6.5 (2.5‐13)
	Group B	25.4 (20‐34.2)	9 (2.3‐23.6)	10 (7‐15.5)	5 (1-11)
	Group C	37 (24‐44.4)	9 (6‐19.2)	13 (9-17)	5 (2-9)

a SCORAD: Scoring Atopic Dermatitis.

b POEM: Patient-Oriented Eczema Measure.

c Group A: participants who did not receive the app.

d Group B: participants who engaged with the application on fewer than 8 days.

e Group C: participants who engaged with the app on 8 or more days.

Multiple regression analysis aimed to explore effects of various independent variables on changes in SCORAD and POEM scores between visits. The independent variables included the prescription of different types of medications at the previous visit, interval since the screening visit, and use of the Atopic App for 8 days or more following the screening visit.

An *F* test was used as a measure of the models’ accuracy on the dataset:

- For the model with SCORAD score as the dependent variable: multiple *R*=0.6282, *R*^2^=0.3947, *F*_6,75_=8.1497, *P*<.001.
- For the model with POEM score as the dependent variable: multiple *R*=0.6731, *R*^2^=0.4531, *F*_6,75_=10.3548, *P*<.001.

The explored models identified statistically significant predictors of SCORAD and POEM score changes:

1. The prescription of topical corticosteroids at the previous visit was a strong predictor of a decrease in both SCORAD and POEM scores (*P*<.001 and *P*<.001, respectively).
2. Engagement with the Atopic App for 8 days or more following the screening visit strongly predicted a decrease in both SCORAD and POEM scores (*P*=.01 and *P*=.04, respectively).
3. The time elapsed since the screening visit significantly predicted an increase in both SCORAD and POEM scores (*P*<.001 and *P*<.001, respectively).
4. Prescriptions of topical calcineurin inhibitors, oral antihistamines, and oral antibiotics were weak and insignificant predictors of SCORAD and POEM score changes.

Most participants experienced seamless use of the installed app, with only four inquiries pertaining to its use arising during the initial week post installation.

## Discussion

### Principal Findings

The number of AD-related mobile apps is increasing [3]. However, only a few of these apps are designed to enable bidirectional communication between patients or caregivers and the app [7]. Most studies on apps usage for AD focus primarily on the acceptability and feasibility of using AD-related mobile apps to assess disease severity or burden of the disease [5,8,9] and all lack supporting evidence, input from clinicians and dermatologists [10]. However, this is a randomized controlled study that provides evidence for the potential impact of engaging caregivers of children with AD on the dynamic changes in AD severity, using an app developed with the aid of 20 dermatologists, allergists, adult patients with AD, and parents of children with AD, and aimed to identify difficulties in management of AD in home settings [6]. The differences in methodology of these studies from this randomized controlled trial study preclude meaningful comparison of our findings to other studies on mobile health apps for AD.

In their analysis of studies on the effectiveness of mobile phone apps in influencing health-related behavior change, Zhao et al [11] concluded that apps’ use for treatment reminders leads to increased overall adherence. More recently, Joergensen et al [12] investigated the effects of requested self-reporting on treatment adherence using memory buttons, with or without a mobile app, and reported improved outcomes in the group that used the mobile app. A recent meta-analysis [3] demonstrated that while mHealth applications significantly improve patients’ quality of life and self-management, they show no significant impact on AD severity (SCORAD). This suggests that the reviewed apps fail to offer comprehensive tools for tracking and addressing clinical symptoms, which may be attributed to limited personalization of treatment plans and lack of real-time communication with health care providers. In a qualitative study, caregivers and health care professionals highlighted key shortcomings in existing apps, such as confusion over treatment, lack of empowerment through education, and limited emotional support [4]. These factors contribute to poor user engagement and adherence, which aligns with findings from a feasibility study [5], where declining weekly interactions with app features over 6 weeks was noted despite initially high engagement with medication reminders and educational content. Maintz et al [13] highlighted a common limitation of AD apps: inadequate interoperability, data exchange, and personalized care. Lack of bidirectional communication remains a key challenge in many existing tools, as they often function as static symptom trackers without the capacity to facilitate ongoing interaction between patients and health care professionals.

The Atopic App addresses the identified limitations in current AD management, such as AI-powered severity assessment, incorporation of personalized action plans, bidirectional communication and sustained engagement strategies.

The minimal clinically important differences (MCIDs) for SCORAD and POEM scores in AD have been established to represent the smallest changes in scores that are perceived as meaningful by patients: the MCID for SCORAD is about 8.7 points and for the POEM about 3.4 points [14]. The reductions in SCORAD and POEM scores observed in our study significantly exceed the established MCID thresholds, reinforcing the effectiveness of the Atopic App as a valuable adjunct tool in managing AD in children. While all groups exhibited reduced disease severity over time, patients who engaged more with the app—specifically, those who used it for 8 days or more between visits—demonstrated a more significant reduction in severity scores. This suggests that repeated exposure to patient-education content and the app’s feedback may lead to better adherence to treatment plans and improved disease management [6]. On the other hand, participants who actively engaged with the app may have been more likely to adhere to treatment plans and recommendations, potentially influencing their improved outcomes. To strengthen causal inferences between app usage and improved outcomes in AD management, future research could use random assignment of app access, independent of participants’ initial engagement levels that would isolate the app’s true impact on treatment adherence and clinical outcomes.

While the reductions in AD severity observed across all study groups could be attributed to various factors such as prescribed treatments, the natural course of the disease, increased awareness and monitoring, or a placebo effect, the results of the multiple regression analyses indicate that these possibilities are highly unlikely. The analyses showed a significant correlation between higher SCORAD and POEM scores and the time period from the screening visit, which suggests that factors other than those mentioned above are at play. Additionally, the prescription of topical corticosteroids at a preceding visit was identified as a strong predictor of decreased SCORAD and POEM scores, highlighting the well-established effectiveness of this treatment. However, the time elapsed since the screening visit was found to be a significant predictor of increased disease severity, emphasizing the need for regular monitoring and intervention. Notably, sustained app engagement was linked to lower disease scores, emphasizing its potential role in AD management. While capturing disease state at exacerbation onset would offer deeper insights, this study primarily focused on longitudinal changes.

### Limitations of this Study

This study’s population exhibited a gender imbalance with a higher proportion of boys compared to girls. While this does not reflect the general population, this pilot study prioritized feasibility and initial impact assessment of the Atopic App. To comprehensively evaluate the app’s effectiveness, a larger study with a balanced gender distribution is warranted.

Possibly, patients who engaged more with the app may have been more proactive in seeking medical advice or adjusting their treatment based on the app’s recommendations, leading to better outcomes.

The predominance of female caregivers in our study highlights the importance of considering gender as a contextual factor, as recommended by the COSMIN (Consensus-Based Standards for the Selection of Health Measurement Instruments) guidelines. Due to our limited sample size, we were unable to perform a formal subgroup analysis by gender. Nevertheless, the high proportion of female participants likely reduces the risk of significant gender-related bias in our findings. Future research involving larger and more diverse populations will be crucial to further examine the influence of gender and other contextual factors on the effectiveness of the app.

### Conclusion

The Atopic App represents a significant advancement in the digital management of AD by offering AI-powered, personalized solutions to the common shortcomings of existing apps, including lack of bidirectional communication, low engagement, and inadequate personalization. By addressing these gaps, the Atopic App provides a more effective, user-centered approach to improving treatment adherence and clinical outcomes in AD, positioning it as a valuable contribution to the field.

Future research directions should explore: (1) sustained use of the app over extended periods to assess its effect on long-term patient outcomes, treatment adherence, and disease control; (2) economic benefits of integrating the Atopic App into clinical workflows, including potential reductions in health care visits and treatment costs; and (3) potential uses of the app’s technology and personalized approach could be adapted for managing other chronic skin conditions.

## Supplementary material

10.2196/60479 Checklist 1 CONSORT-eHEALTH checklist (V 1.6.1). CONSORT: Consolidated Standards of Reporting Trials

## Abbreviations

AD: atopic dermatitis
AI: artificial intelligence
COSMIN: Consensus-Based Standards for the Selection of Health Measurement Instruments
MCID: minimal clinically important difference
POEM: Patient-Oriented Eczema Measure
SCORAD: Scoring Atopic Dermatitis
